# Algorithms for the executable programs planning on supercomputers

**DOI:** 10.1371/journal.pone.0275099

**Published:** 2022-09-26

**Authors:** Abdullah M. Algashami

**Affiliations:** Department of Computer Science and Information, College of Science at Zulfi, Majmaah University, Al-Majmaah, Saudi Arabia; Nottingham Trent University School of Science and Technology, UNITED KINGDOM

## Abstract

This research dealt with the problem of scheduling applied to the supercomputer’s execution. The goal is to develop an appreciated algorithm that schedules a group of several programs characterized by their time consuming very high on different supercomputers searching for an efficient assignment of the total running time. This efficient assignment grantees the fair load distribution of the execution on the supercomputers. The essential goal of this research is to propose several algorithms that can ensure the load balancing of the execution of all programs. In this research, all supercomputers are assumed to have the same hardware characteristics. The main objective is to minimize the gap between the total running time of the supercomputers. This minimization of the gap encompasses the development of novel solutions giving planning of the executable programs. Different algorithms are presented to minimize the gap in running time. The experimental study proves that the developed algorithms are efficient in terms of performance evaluation and running time. A comparison between the presented algorithms is discussed through different classes of instances where in total the number of instances reached 630. The experiments show that the efficient algorithm is the best-programs choice algorithm. Indeed, this algorithm reached the percentage of 72.86%, an average running time of 0.0121, and a gap value of 0.0545.

## 1 Introduction

This paper focuses to develop an algorithm for the problem related to the scheduling of the execution programs by supercomputers. Different programs are received by the administrator to be scheduled on the available supercomputers. Each program has its own executing time. The supercomputers must be operated in the same way and with the same use of time. This is can be reached by an appropriate algorithm that can schedule the received programs with a fair way distribution. Otherwise, when fairness is not applicable, one supercomputer can be more exploited than another one.

The problem presented in this paper can be defined as follows. Suppose that there are several executable programs. These programs need a big amount of memory and a robust recourses like processor to ensure the accomplishment of the total execution. Each program is described by its own running time. The objective is concerned with finding a method that give a schedule for these executable programs guaranteeing a fair distribution in term of running time. There is no previous research in the literature that studied the proposed problem. However, many research works can be referred to the load balancing problem.

The load balancing treated the budget distribution is developed in [1]. A mathematical formulation was proposed to give an objective function for the problem. This formulation applied the minimization of the gap in the cumulative income between different regions. Three algorithms were treated to solve the latter problem. The randomization procedure is used as the first approximate solution. The second one is an iterative algorithm. The last one is the moving of the probabilistic values. In the same context, another work treated the project assignment was studied in [2]. In this work, the authors formulated the problem by giving a new objective function. Indeed, a minimization of the maximum revenue was studied. In this latter research, different algorithms were developed. These algorithms utilize the dispatching rules method and the multi-fit method. Different groups of instances are tested to measure the efficacy of the given algorithms.

In [3], the authors developed solutions regarding the cloud repository problem using the load balancing procedures. This paper focus on the issues in relation to good operation of storage servers in the cloud environment. The main objective of this research will contribute in handling several challenges concerning the load balancing in the cloud environment. Though analyzing the researches discussing topics in this field, in this paper 2 distributed load-balancing procedures. Similar work can be cited as [4]. A review regarding the load-balancing approaches in cloud context is proposed in [5].

Load-balancing dispatches the workload through several nodes to obtain better results when exploited the system. Different load balancing procedures occur to reach better resource exploitation. In [6], authors developed a discussions of load balancing procedures. In addition, the authors in the latter paper, give a comparison between the proposed algorithms on the basis of different indicators like mean no-waiting time, processing time, and data cost time.

The load balancing procedures are used in literature in the domain of the projects and budgets distribution. Indeed, in [7], several lower bounds were proposed, different heuristics, and a exact method for the project distribution were dedicated to propose solution. In this latter paper, the objective function is proposed as a new one compared with the one given in [2]. In [1], the author proposed three heuristics to solve the problem of the projects revenues assignment problem. In the same context, in [8] the authors proposed an exact algorithm for the budget scheduling problem. Different heuristics and algorithms were proposed to be used in the exact approach. The load balancing problem is studied and applied on many domains in literature.

The load balancing used on the storage spaces is utilized in [9]. In fact, several algorithms were developed. These algorithms are used in different by the dispatching rules approach. A comparison between the proposed algorithms are discussed.

A load balancing procedures regarding the supercomputers are treated in different previous works. A periodic load balancing procedure are studied in [10]. The load balancing regarding the message-passing in supercomputer were treated in [11].

On the other hand, the utilization of the load balancing is adopted in the network field. Indeed, several algorithms were developed to find an acceptable solution that ensuring the equity transmission of the give data [12].

Another field that the load balancing is applied, is the aircraft field [13]. Authors proposed several lower bounds regarding the load balancing applied on the gas turbine field. These lower bounds are based basically on the iterative approach, subset sum problem and the knapsack problem. In [14], the authors treated the same problem studied in [13] by using the randomization method.

In the domain of health care, several clustering algorithms which are used two sets is the best algorithm with 96% for the small scale instances and 98% for the big instances. The novelty of this research is the utilization of the dispatching rules by different modifications; randomized method, clustering approach; probability application algorithm, and multi-start algorithm to schedule quality reports to available physicians. The objective is to guarantee the fair assignment of the number of papers workload [15].

Authors in [16] developed a cost model related to the correctness of the load imbalance. This model offers discussions of the efficiency of load balancing procedures in any particular imbalance case. The developed process, in this latter paper, correctly selects the algorithm that carry out the lowest running time in up to 96% of the total cases, and may accomplish a 19% profit over selecting a single balancing procedure for all generated cases.

Utilizing loop parallelism is obviously most critical in accomplishing high system and routine efficiency. Because of the clarity of this method, guided self-scheduling is specifically adapted for execution on real parallel machines. This approach accomplishes concurrently the two most significant goals: load balancing and extremely low synchronization overhead. For particular types of repeating the results prove analytically that guided self-scheduling utilize minimal overhead and accomplishes optimal schedules. Two other interesting properties of this approach are its insensitivity to the first processor configuration (in time) and its parameterized nature which enables us to tune it for different systems [17].

In the industrial domain, the load balancing algorithms are used in [18] to ensure a better utilization of the machines and guarantee an equity use of machines. In the same context, the authors in [19] solve the equity distribution of the jobs on the machines by the multi-start algorithms applying the probabilistic method.

A dynamic balancing algorithm assumes the low effects of two main elements of the system which related to job behavior and the general state of the system, i.e., load balancing procedure using the actual status of the system. The establishment of an efficacious dynamic load balancing procedure encompasses several essential issues: load cost, load standard comparison, assessment indicators, system stability, amount of data exchanged between nodes, job resource required, job’s chosen for transfer, remote nodes chosen, and more [20].

In addition, dispatching the persons into vehicle ti ensure an equity distribution of these persons on the available parking is proposed in [21]. Recently, authors in [22] proposed novel algorithms to solve the parking managment. Several researches have been uploaded in the literature in order to cover some aspects in relation to the execution period and the gap valuation, which have been used to explore the assessment of the development procedures. Analyzing the gathered experiments shows a good indication in the assessment behavior of the proposed algorithm. In addition, it shows the proposed algorithm can narrow the gap in issues in relation to gap and time calculation in the developed researches. The *MR* heuristic reached an exceptional assessment results compared this result with the best algorithms proposed in [21]. The *MR* heuristic reached a percentage of 96%, a gap of 0.02, and a running time of 0.007s.

The usage of the supercomputers is largely referred in the literature. In fact, the utilization of the scheduling on the supercomputers filed may be cited to the different works. In [23], the authors studied the resource management through the usage of the supercomputers by an energy-performance procedure. The parallelism of the processing in the supercomputers is proposed by [24].

In [25], authors considered the methods of optimal load balancing and the storage requirements of algorithms. Other algorithms proposed in [26] can be utilized in future work to enhance the proposed algorithms. A mathematical model for scheduling activities while there are priorities between devices are proposed in [27]. In [28], authors treated the scheduling algorithms into networks. These algorithms can be exploited to give a new solutions for the presented problem. In [29], authors developed algorithms for the category constraint into network based on scheduling problem. Authors in [30] developed algorithms for the read frequency of data. These algorithms may be exploited on and adopted for the presented problem. A recent similar work for the latter paper are proposed in [31].

In this paper, a mathematical model of the presented problem is proposed. In addition, many algorithms were proposed to manage the studied problem. The problem can be defined as follows. Several programs characterized by its estimated execution time need to be ran by several supercomputers. All the supercomputers are assumed to be characterized by the same criteria as the hardware. The goal is to search for an efficient algorithm to schedule these programs to the available supercomputers. This is can be mathematically written as the load balancing of programs to supercomputers.

The rest of the paper is structured as follows. Section 2 presents the justification and motivation to work the studied problem. In section 3, the problem definition is described. Section 4 presents the research method and design. The developed algorithms to solve the presented problem are detailed in Section 5. The experimental results and discussions are analysed in Section 6. Section 7 is reserved for the conclusions and perspectives.

## 2 Justification and motivation

The presented problem may be defined as follows. Suppose that there are a set of several programs characterized by its estimated running time. This set of programs need to be executed by many available supercomputers. The set of programs is homogeneous. This is means that all the programs in the set have the same hardware characteristics. In addition, these programs are supposed to consume more resources in memory and time execution. Consequently, it is primordial to seek an efficient way to schedule the given programs on the available supercomputers. This may be mathematically written as the equity assignment of programs to the supercomputers. The goal of this paper is to concept and design an algorithm that may minimize the execution time gap between all supercomputers. The first phase toward achieving the goal of this paper is to formulate mathematically the proposed problem. After that, this mathematical formulation is utilized to develop different algorithms to solve the presented problem. This is constitute the second phase. A detailed discussions and explanation of these two phases are presented in this paper.

## 3 Problem definition

Table 1 gives an overview for all variable notations and definitions used in the paper.

**Table 1:** Variable notations and definitions.

Variable	Definition
*Pr*	Set of programs that will be executed by the different supercomputers
*n*_*pr*_	Number of programs delivered by the administrator
*Sr*	Set of supercomputers
*n*_*su*_	Number of supercomputers
*p*	The program index
*Pr*_*p*_	The program number *p*
*i*	The supercomputer index
*Su*_*i*_	The supercomputer number *i*
*e*_*p*_	The estimated execution time for the program *p*
*ct*_*p*_	Cumulative execution time when *p* is scheduled
*Rt*_*i*_	The total execution time for each *i* after accomplishing the execution of all programs
*Rt*_*min*_	$\min_{i=\{1,\cdots ,n_{s}\}}Rt_{i}$
*A*_*b*_	The minimum gap value reached after finishing the workload of all algorithms
*A*	The gap value given by the presented algorithm
$Gp=\frac{A-A_{b}}{A}$	The gap between the minimum value and the presented one
*Time*	Average running time in seconds. In Tables “*” means that the execution time is less than 0.0001 s
*Pcg*	Percentage of programs where *A*_*b*_ = *A* among all tested instances

Example 1 explains the presented problem using all above definitions.

**Example 1** *Suppose that n*_*su*_ = 2 *and n*_*pr*_ = 7. *The e*_*p*_ *value for each program is illustrated in* Table 2.

**Table 2:** The estimated running time for each program.

*Pr*_*p*_	*Pr*_1_	*Pr*_2_	*Pr*_3_	*Pr*_4_	*Pr*_5_	*Pr*_6_	*Pr*_7_
*e*_*p*_	261	291	231	371	491	201	311

**Fig 1:**
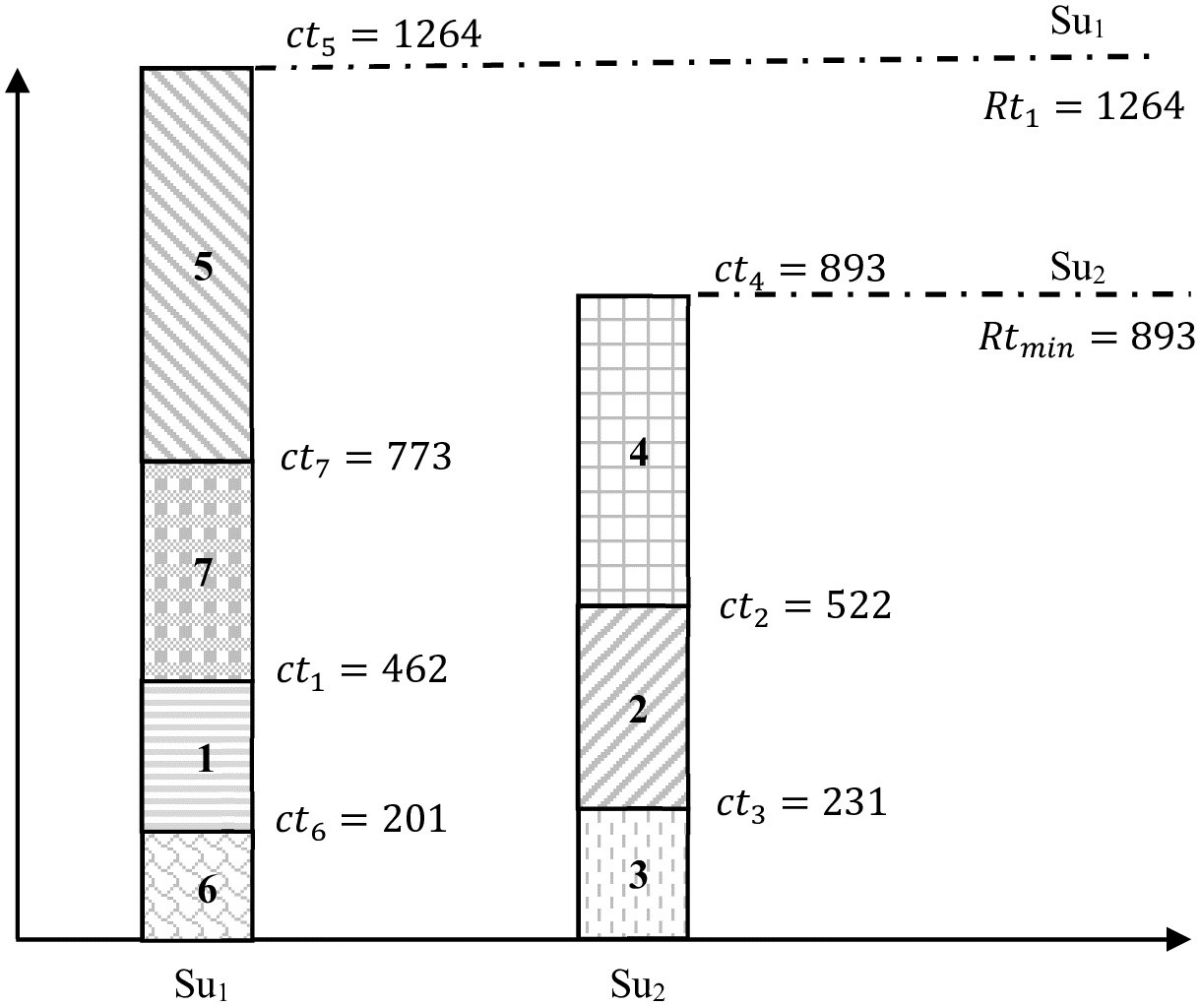
Shortest execution time schedule for Example 1.

*Now, an algorithm is ran to assign the programs detailed in* Table 2 *to the available supercomputers. This algorithm is the shortest execution time algorithm, the obtained schedule is illustrated in* Fig 1. *It is evident to see that programs* {1, 5, 6, 7} *are executed by Su*_1_ *and programs* {2, 3, 4} *are executed by Su*_2_.

Fig 1 *shows that Su*_1_ *has a total execution time of 1264. Moreover, Su*_2_ *has a total execution time of 893. Accordingly, the execution time gap between Su*_1_ *and Su*_2_ *is Rt*_1_ − *Rt*_*min*_ = 1264 − 893 = 371. *The goal is to conecpt and design an algorithm that can reduce the returned gap between supercomputers. In fact, for Example 1 another schedule must be given with a better solution comparing with schedule 1. This is means that an algorithm giving a gap less than 371*.

**Fig 2:**
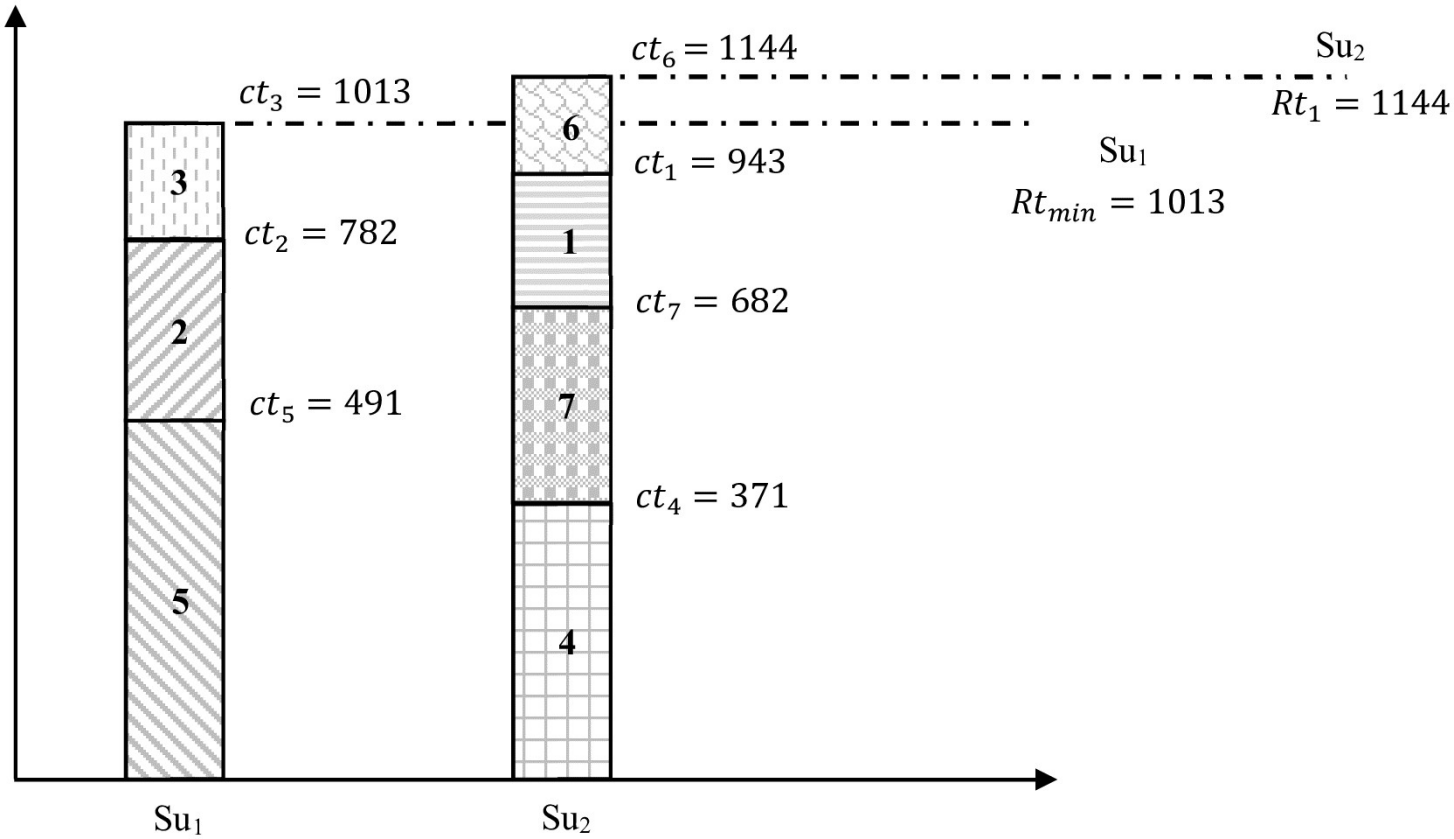
Longest execution time schedule for Example 2.

**Example 2** *For this example, the instance detailed in* Table 2 *is examined. Calling the longest execution time algorithm, the result given the schedule is illustrated in* Fig 2. *It is easy to see that programs* {2, 3, 5} *are executed by Su*_1_ *and programs* {1, 4, 6, 7} *are executed by Su*_2_.

Fig 2 *shows that Su*_1_ *has a total execution time of 1013. Moreover, Su*_2_ *has a total execution time of 1144. Thus, the execution time gap for Su*_1_ *and Su*_2_ *is Rt*_2_ − *Rt*_*min*_ = 1144 − 1013 = 131. *For this example, the schedule 2 gives a better results than schedule 1*.

In general, facing on different supercomputers, an indicator must be determined to evaluate the gap of the algorithm that searching the load balancing. Eq 1 represent the gap of the execution. This gap is calculated between the supercomputer that having the minimum total execution time and all others supercomputers. This gap must be minimized to guarantee the load balancing. Hereafter, this gap is denoted by *Grt*.

$$\begin{array}{c} Grt=\sum_{i=1}^{n_{s}}\left(Rt_{i}-Rt_{min}\right). \end{array}$$ (1)

**Proposition 1** *Based on* Eq 1, *the gap Grt may be formulated such that in* Eq 2.

$$\begin{array}{c} Grt=\sum_{i=1}^{n_{s}}Rt_{i}-n_{s}Rt_{min}. \end{array}$$ (2)

**Proof 1** $Grt=\sum_{i=1}^{n_{s}}\left(Rt_{i}-Rt_{min}\right)=\sum_{i=1}^{n_{s}}Rt_{i}-\sum_{i=1}^{n_{s}}Rt_{min}$. *It is clear to see that* $\sum_{i=1}^{n_{s}}Rt_{min}=n_{s}Rt_{min}$. *Thus, the* Eq 2 *is obtained*.

## 4 Research method and design

**Fig 3:**
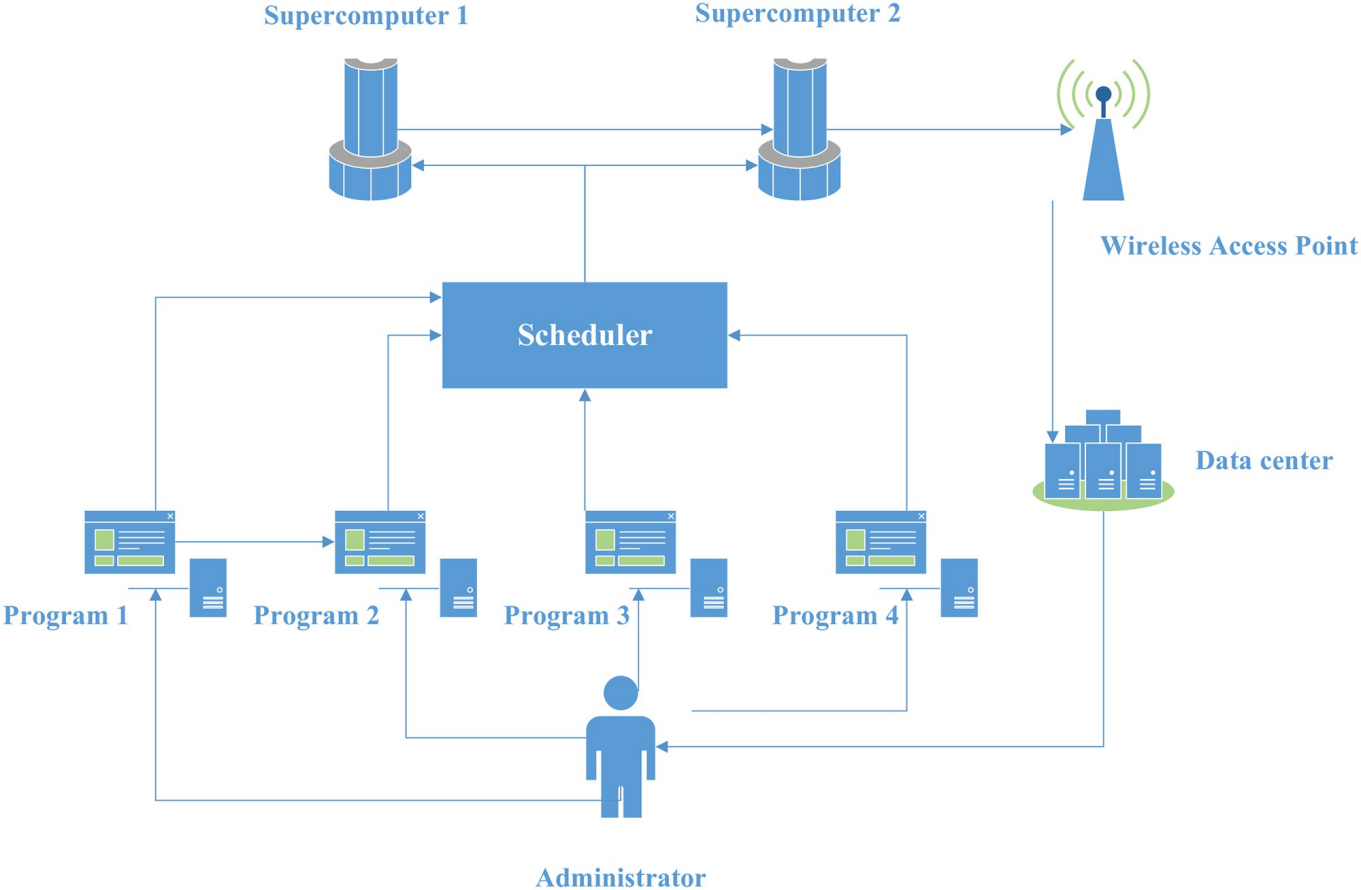
4-programs and 2-supercomputers example for the design model.

In this section, six components are proposed for the proposed model. To more understand the model proposed in this research, an example is given of two supercomputers and four programs as shown in Fig 3. The first component is “supercomputer”. For the example given in This component contain the supercomputer. The “data center” is a component that can receive the results of the executed programs by the supercomputers. In addition, this component is responsible to send all new programs to be executed to the administrator. The component which guarantee an efficient transmission between the supercomputers and the data center is the “Wireless Access Point”. The “administrator” is the component represented by the user that having all access authorization and can decide for choosing the programs sent by the data center. Finally, the component “scheduler” is responsible to apply the developed algorithms to propose a solution regarding the good distribution of programs to the different supercomputers. The main component in this research work is the “scheduler”.

In this paper, a new design of the studied problem regarding the planning of the executable program on the available supercomputers is proposed as illustrated in Fig 3. The proposed components are focalized on the scheduler one. The scheduler is responsible to call all the algorithms to solve the scheduling problem and decide which program must be executed on the fixed supercomputer. Several variants of probabilistic method are proposed. In general, using a probabilistic method and the randomization approach give an efficient approximate solution for the scheduling solution problem. It is important to notice that the developed problem is NP-hard one. This is confirm that a good approximate solution represent a great archive for the studied problem.

**Fig 4:**
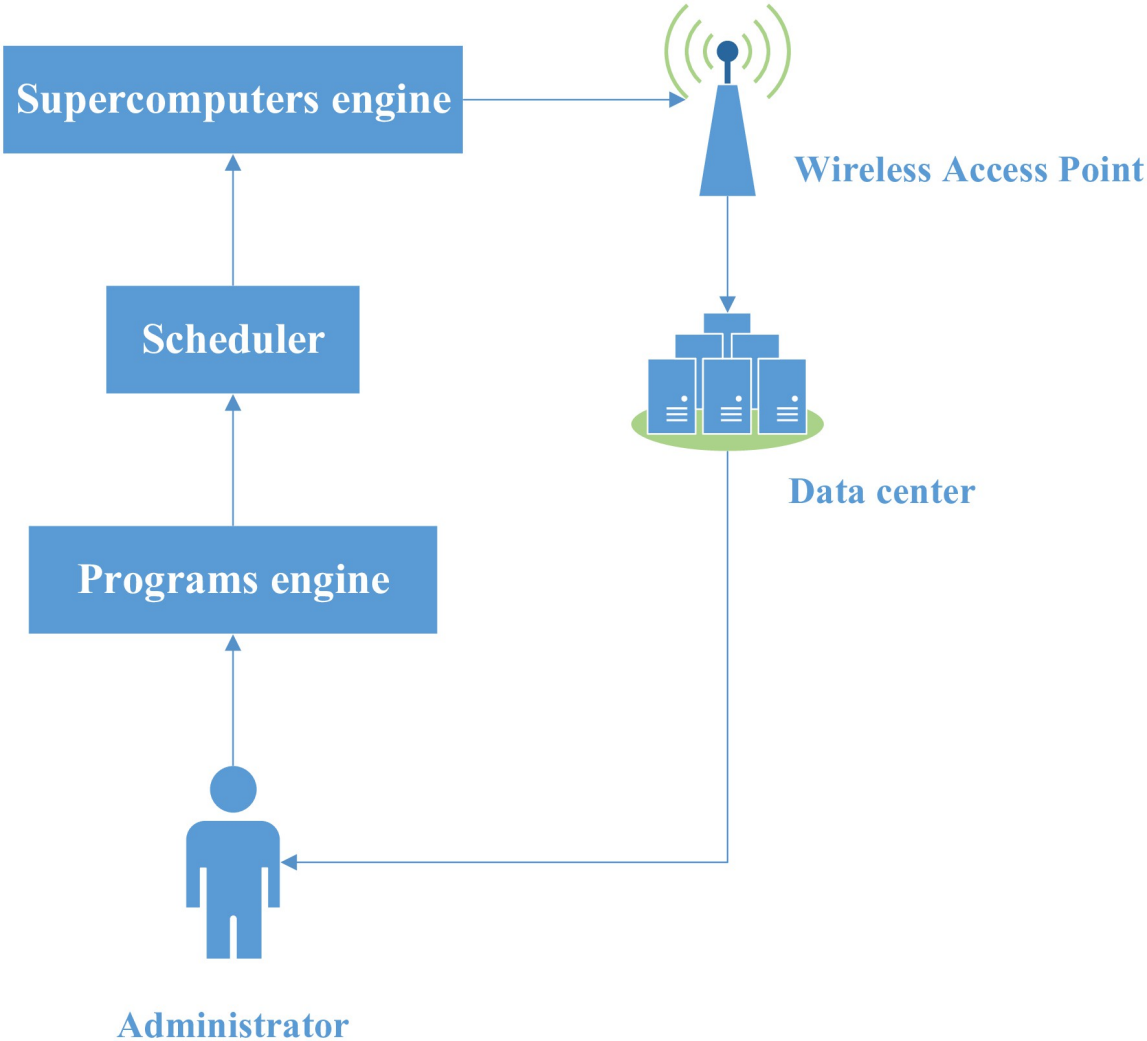
Design of the supercomputers-program model.

Based on the example of 4-programs and 2-supercomputers shown in Fig 3, a generalization of the model as shown in Fig 4 can be illustrated. The general model is composed by five fundamental components: Supercomputers engine, scheduler, Programs engine, wireless access point, and data center.

## 5 Developed algorithms

In this section, seven proposed algorithms will be presented and detailed. Indeed, each algorithm will be explained and a pseudo-code will be illustrated for some algorithms to explain the functionality of these algorithms. The complexity of each algorithm is given.

### 5.1 Longest execution time algorithm

A dispatching rule method is applied for the longest-running time algorithm (*LTA*). Firstly, all programs are listed in the non-increasing order of its execution time. Next, the programs that have the longest execution time will be executed by the supercomputer that has the minimum *ct*_*p*_ and so on until completing all the workload.

### 5.2 Shortest execution time algorithm

A dispatching rule method is applied for the longest-running time algorithm (*STA*). Firstly, all programs are listed in the increasing order of its execution time. Next, the programs that have the longest execution time will be scheduled on the supercomputer that has the minimum *ct*_*p*_, and so on until completing all workload.

### 5.3 Programs choosing probabilistic algorithm (*PCP*)

This algorithm applies the probabilistic approach. The first program which will be chosen to be ran by the supercomputer is picked by a probability *β*. After that, the second program will be picked among the remaining programs applying the same probability *β* and so on until completing all workload. In the implementation code, the calculation of the probability is determined as follows. In the first, a number *h* is generated and returned randomly in [1, *n*_*s*_]. Now, *Pr*_*h*_ is selected and executed by the available supercomputer. The set *Pr* will be updated by removing *Pr*_*h*_ from this set. So, *Pr* = *Pr* \ *Pr*_*h*_. This procedure is repeated *Lmt* = 1000 times and the minimum value of the returned gap *Grt* will be stored.

Hereafter, *Rad*(*x*, *y*) denoted the function that give a random number in [*x*, *y*]. *SLg*(*r*, *z*) is the function that schedules *Pr*_*r*_ on *Su*_*z*_.

This algorithm is *PCP* and all details are illustrated in Algorithm 1. The complexity of *PCP* algorithm is *O*(*n*^2^).

**Algorithm 1** *PCP* algorithm

1: **for** (*v* = 1 to *Lmt*) **do**

2:  Fix *x* = *n*_*su*_

3:  **while** (*x* ≥ 1) **do**

4:   Fix *k* = *Rad*(1, *x*)

5:   Available supercomputer is *Su*_*z*_

6:   Call *SLg*(*k*, *Su*_*z*_)

7:   Fix *x*− −

8:  **end while**

9:  Determine *Grt*_*v*_

10: **end for**

11: Determine $Grt_{}=\underset{1\leq v\leq Lmt}{\text{min}}Grt_{v}$

### 5.4 Non-decreasing-order-programs choosing probability algorithm

This algorithm applies the probability-approach as detailed in the Subsection 5.3. In the first, the programs is sorted in the non-decreasing order of its estimated execution time. The selected program that can be ran by the free supercomputer is picked by a probability *γ*. This instruction will be repeated *Lmt* = 1000 times and the minimum obtained gap *Grt* will be stored. Hereafter, *ICG*(*m*) represent the function that sort *Pr*_*m*_ in the non-decreasing order of its estimated execution time. The complexity of *NCP* algorithm is *O*(*n*^2^).

This algorithm is denoted by *NCP*. The instructions of *NCP* are illustrated in Algorithm 2.

**Algorithm 2** *NCP* algorithm

1: Call *ICG*(*Pr*)

2: **for** (*v* = 1 to *Lmt*) **do**

3:  Fix *x* = *n*_*su*_

4:  **while** (*x* > 0) **do**

5:   Fix *k* = *Rad*(1, *x*)

6:   Available supercomputer is *Su*_*z*_

7:   Call *SLg*(*k*, *Su*_*z*_)

8:   Fix *x*− −

9:  **end while**

10:  Determine *Grt*_*v*_

11: **end for**

12: Determine $Grt_{}=\underset{1\leq v\leq Lmt}{\text{min}}Grt_{v}$

### 5.5 Decreasing-order-choosing probabilistic algorithm

This algorithm applies the randomization approach as detailed in the Subsection 5.3. In the first, the programs is sorted in the non-increasing order of its *e*_*p*_. The picked program which will be ran by the free supercomputer is chosen by a probability *γ*. This instruction is repeated *Lmt* = 1000 times. The minimum gap will be stored. Denoted by *DRG*(*Pm*) the procedure that sort the programs *Pm* in the non-increasing order of its estimated execution time. The complexity of *DCP* algorithm is *O*(*n*^2^).

This algorithm is *DCP*. The details of *DCP* are illustrated in Algorithm 3.

**Algorithm 3** *DCP* algorithm

1: Fix *DRG*(*Pr*)

2: **for** (*v* = 1 to *Lmt*) **do**

3:  Fix *x* = *n*_*su*_

4:  **while** (*x* > 0) **do**

5:   Fix *k* = *Rad*(1, *x*)

6:   Available supercomputer is *Su*_*z*_

7:   Call *SLg*(*k*, *Su*_*z*_)

8:   Fix *x*− −

9:  **end while**

10:  Determine *Grt*_*v*_

11: end for

12: Determine $Grt=\underset{1\leq v\leq Lmt}{\text{min}}Grt_{v}$

13: Return *Grt*

### 4.6 Three-variant-programs choosing probabilistic algorithm

This algorithm applies the probabilistic approach as detailed in the above subsections. In first, *PCP* algorithm is called. The solution obtained by *PCP* is Denoted by *Grt*1. After that, the *NCP* algorithm is called. Denoted by *Grt*2 the returned solution. Finally, *DSP* algorithm is called and denoted by *Grt*3 the returned solution. The best solution between *Grt*1, *Grt*2 and *Grt*3 is picked. The complexity of *TSP* is *O*(*n*^2^). Denoted by *PCP*(*Pm*), *NCP*(*Pm*), and *DCP*(*Pm*) the functions calling the algorithms *PCP*, *NCP*, and *DCP*, respectively.

This algorithm is *TVP*. The details of *TVP* are illustrated in Algorithm 4.

**Algorithm 4** *TVP* algorithm

1: Call *PCP*(*Pm*)

2: Determine *Grt*1.

3: Call *NCP*(*Pr*)

4: Determine *Grt*2.

5: Call *DCP*(*Pr*)

6: Determine *Grt*3.

7: Determine *Grt* = min(*Grt*1, *Grt*2, *Grt*3)

8: Return *Grt*

### 5.7 Best-programs choosing algorithm

This algorithm use *LRT* and *TVP* algorithms. Indeed, *LRT* and *TVP* algorithms are called separately and the best result is selected. This algorithm is *BPC*.

## 6 Experimental results and discussions

Many indicators are given to assess the efficiency of the presented algorithms. Through these indicators, a comparison between the proposed algorithms are discussed. All proposed algorithms were implemented in C++. The computer executing all the developed code is an Intel(R) Core (TM) i5-3337U CPU and the operating system is Windows 10.

### 6.1 Instances and tests

Three classes are proposed in the subsection “Instances” to measure the efficiency of the presented algorithms and three indicators are presented in the subsection “Tests”.

#### 6.1.1 Instances

Different instances are tested and experimented. The types of classes applied in this paper are the uniform distribution which is denoted by *UN*[*x*_1_, *x*_2_]. The comparative study between the algorithms and the manner that the instances are generated are inspired from the study [32].

The *e*_*p*_ values will be as:

- Class A: *x*_1_ = 1 and *x*_2_ = 100, *e*_*p*_ ∈ *UN*[1, 100].
- Class B: *x*_1_ = 10 and *x*_2_ = 150, *e*_*p*_ ∈ *UN*[10, 150].
- Class C: *x*_1_ = 100 and *x*_2_ = 500, *e*_*p*_ ∈ *UN*[100, 500].

The permutation of the pair (*n*_*pr*_, *n*_*su*_) discussed in this experimental results are listed as follows. The small scale is for *n*_*pr*_ = {10, 25, 30} the number of supercomputers is *n*_*su*_ = {4, 5, 6}. The big scale is for *n*_*pr*_ = {50, 60, 80, 100} the number of supercomputers is *n*_*su*_ = {5, 6, 10, 12}

For each pair (*n*_*pr*_, *n*_*su*_) and each class, 10 instances were tested. The total generated instances is 630.

#### 6.1.2 Tests

The indicators *G*_*b*_, *Time*, and *Per* used to asses the algorithms are defined in Table 1.

**Fig 5:**
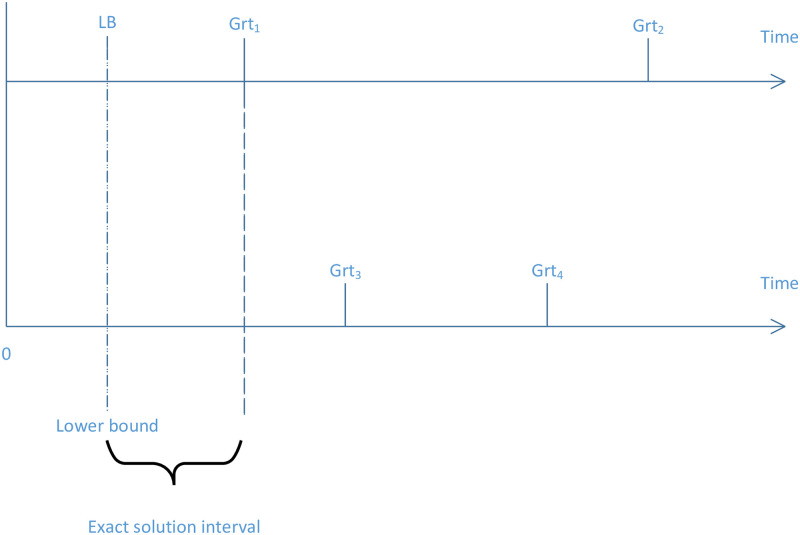
Performance test measurement.

Fig 5 represented the performance test measurement of the proposed algorithms. In this latter figure, it is supposed that there are four different values of the *Grt* obtained by four different algorithms. These values are *Grt*_1_, *Grt*_2_, *Grt*_3_ and *Grt*_4_. It is clear to see that *Grt*_1_ is better than *Grt*_2_, *Grt*_3_ and *Grt*_4_ because *Grt*_1_ is the minimum value and the objective is to minimize *Grt*. The value *LB* in Fig 5 represented the value of the lower bound for the studied problem. In general, the exact solution is in *LB*, *Grt*, with *Grt* is the value obtained by any algorithm. The closest value of *Grt* to *LB* is reached by *Grt*_1_. This is meaning the interval [*LB*, *Grt*_1_] which represent the exact solution interval is the smallest interval comparing when choosing *Grt*_2_, *Grt*_3_ and *Grt*_4_. This is prove that the choice of *G*_*b*_ value to asses the performance of the developed algorithms.

### 6.2 Results

Table 3 illustrated the overview of all algorithms. The variation of *Per*, *G*_*b*_, and *Time* are presented in this latter table. The best algorithm that have the minimum gap is *BPC* reaching a percentage of 72.86%, an average gap of 0.0545 and average execution time of 0.0121 s. The second best algorithm is *TVP* reaching a *Pcg* value of 45.08%, a *Gp* value of 0.1944, and a *Time* value of 0.0121 s. The *STA* never obtained a minimum gap value.

**Table 3:** Overview of all proposed algorithms.

	*LTA*	*STA*	*PCP*	*NCP*	*DCP*	*TVP*	*BPC*
*Pcg*	38.10%	0.00%	25.87%	25.40%	25.56%	45.08%	72.86%
*Gp*	0.3508	0.7626	0.2877	0.2924	0.2937	0.1944	0.0545
*Time*	*	*	0.0041	0.0042	0.0041	0.0121	0.0121

Table 4 shows the *Gp* values variation for all proposed algorithms according to the number of programs. This table displays that the best *Gp* value of < 0.0001 is reached by *TVP* and *BPC* where *n*_*pr*_ = 10. On other hand, a maximum *Gp* value of 0.8905 is reordered by *SRT* where *n*_*pr*_ = 30. For *n*_*pr*_ = 100, the best *Gp* value of 0.0531 is recorded by *BPC* and a maximum *Gp* value of 0.7803 is recorded by *STA*.

**Table 4:** The *Gp* values variation for all proposed algorithms according to the number of programs.

*n*_*pr*_	*LTA*	*STA*	*PCP*	*NCP*	*DCP*	*TVP*	*BPC*
10	0.1954	0.4983	0.0016	0.0103	0.0117	0.0000	0.0000
25	0.4607	0.8321	0.3165	0.3183	0.2746	0.1212	0.0836
30	0.5422	0.8905	0.3840	0.3897	0.4094	0.2876	0.1072
50	0.3501	0.7428	0.2369	0.2648	0.2638	0.1337	0.0557
60	0.0181	0.8118	0.5773	0.5610	0.5652	0.5141	0.0050
80	0.5652	0.7824	0.1805	0.1634	0.2055	0.0771	0.0771
100	0.3242	0.7803	0.3173	0.3392	0.3254	0.2269	0.0531

Table 5 shows the *Gp* values for the proposed algorithms when *n*_*su*_ according to the number of supercomputers. This table displays that the best *Gp* value of 0.0263 is reached by *BPC* where *n*_*su*_ = 14. On other hand, a maximum *Gp* value of 0.8817 is returned by *SRT* where *n*_*su*_ = 4. Where *n*_*su*_ = 14, a maximum *Gp* value of 0.6952 is returned by *STA*.

**Table 5:** The *Gp* values for all algorithms when *n*_*su*_ according to the number of supercomputers.

*n*_*su*_	*LTA*	*STA*	*PCP*	*NCP*	*DCP*	*TVP*	*BPC*
4	0.7875	0.8817	0.2524	0.2733	0.2631	0.1240	0.1240
6	0.4217	0.8021	0.2921	0.3103	0.3137	0.1773	0.0673
8	0.2625	0.6156	0.1363	0.1510	0.1413	0.0784	0.0413
12	0.1791	0.7549	0.3624	0.3488	0.3612	0.2895	0.0305
14	0.2374	0.6952	0.2501	0.2467	0.2424	0.1735	0.0263

Table 6 illustrates the *Gp* values for all algorithms and for each class.

**Table 6:** The *Gp* values for all algorithms and for each class.

*Class*	*LTA*	*STA*	*PCP*	*NCP*	*DCP*	*TVP*	*BPC*
1	0.3582	0.7828	0.3010	0.3072	0.2875	0.1989	0.0545
2	0.3689	0.7014	0.2679	0.2764	0.2802	0.1927	0.0586
3	0.3254	0.8035	0.2943	0.2935	0.3132	0.1915	0.0506

Table 7 shows the *Time* variation for all proposed algorithms when *n*_*pr*_ changes. For *BPC* algorithm, the *Time* values increases when *n*_*pr*_ increase. In addition, the minimum *Time* value of 0.0027 s is returned where *n*_*pr*_ = 10 and the maximum *Time* value of 0.0236 s is returned where *n*_*pr*_ = 100.

**Table 7:** The *Time* variation for all proposed algorithms when *n*_*pr*_ changes.

*n*_*pr*_	*LTA*	*STA*	*PCP*	*NCP*	*DCP*	*TVP*	*BPC*
10	*	*	0.0008	0.0012	0.0010	0.0027	0.0027
25	*	*	0.0018	0.0020	0.0020	0.0058	0.0058
30	*	*	0.0024	0.0024	0.0022	0.0061	0.0061
50	*	*	0.0045	0.0044	0.0046	0.0123	0.0123
60	*	*	0.0049	0.0048	0.0048	0.0150	0.0150
80	*	*	0.0066	0.0065	0.0063	0.0195	0.0196
100	*	*	0.0079	0.0083	0.0076	0.0236	0.0236

Table 8 shows the *Time* variation for all proposed algorithms when *n*_*su*_ changes.

**Table 8:** The *Time* variation for all proposed algorithms when *n*_*su*_ changes.

*n*_*su*_	*LTA*	*STA*	*PCP*	*NCP*	*DCP*	*TVP*	*BPC*
4	*	*	0.0014	0.0016	0.0016	0.0042	0.0042
6	*	*	0.0038	0.0038	0.0039	0.0108	0.0108
8	*	*	0.0021	0.0023	0.0020	0.0052	0.0052
12	*	*	0.0063	0.0065	0.0060	0.0192	0.0192
14	*	*	0.0065	0.0067	0.0064	0.0198	0.0198

**Fig 6:**
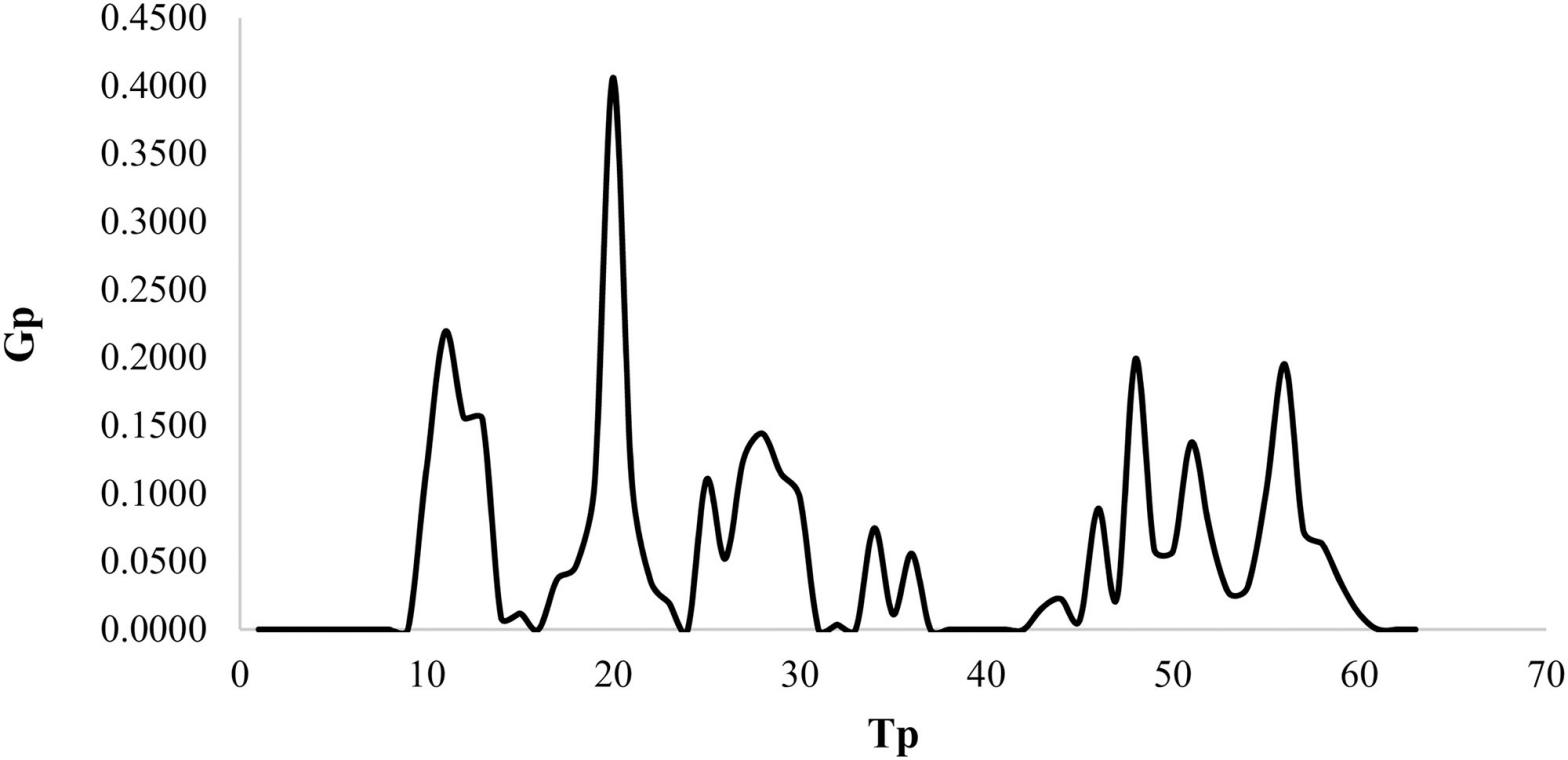
The *Gp* values variation when *Tp* changes for algorithm *BPC*.

Each triple (*n*_*pr*_, *n*_*su*_, *class*) will be denotes by *Tp*. The values of *n*_*pr*_ are {10, 25, 30, 50, 60, 80, 100} and the values of *n*_*su*_ are {4, 6, 8, 12, 14}. Three classes are proposed. So, in total 63 values of *Tp* are presented. Fig 6 shows the *Gp* values variation when *Tp* changes for *BPC*.

**Fig 7:**
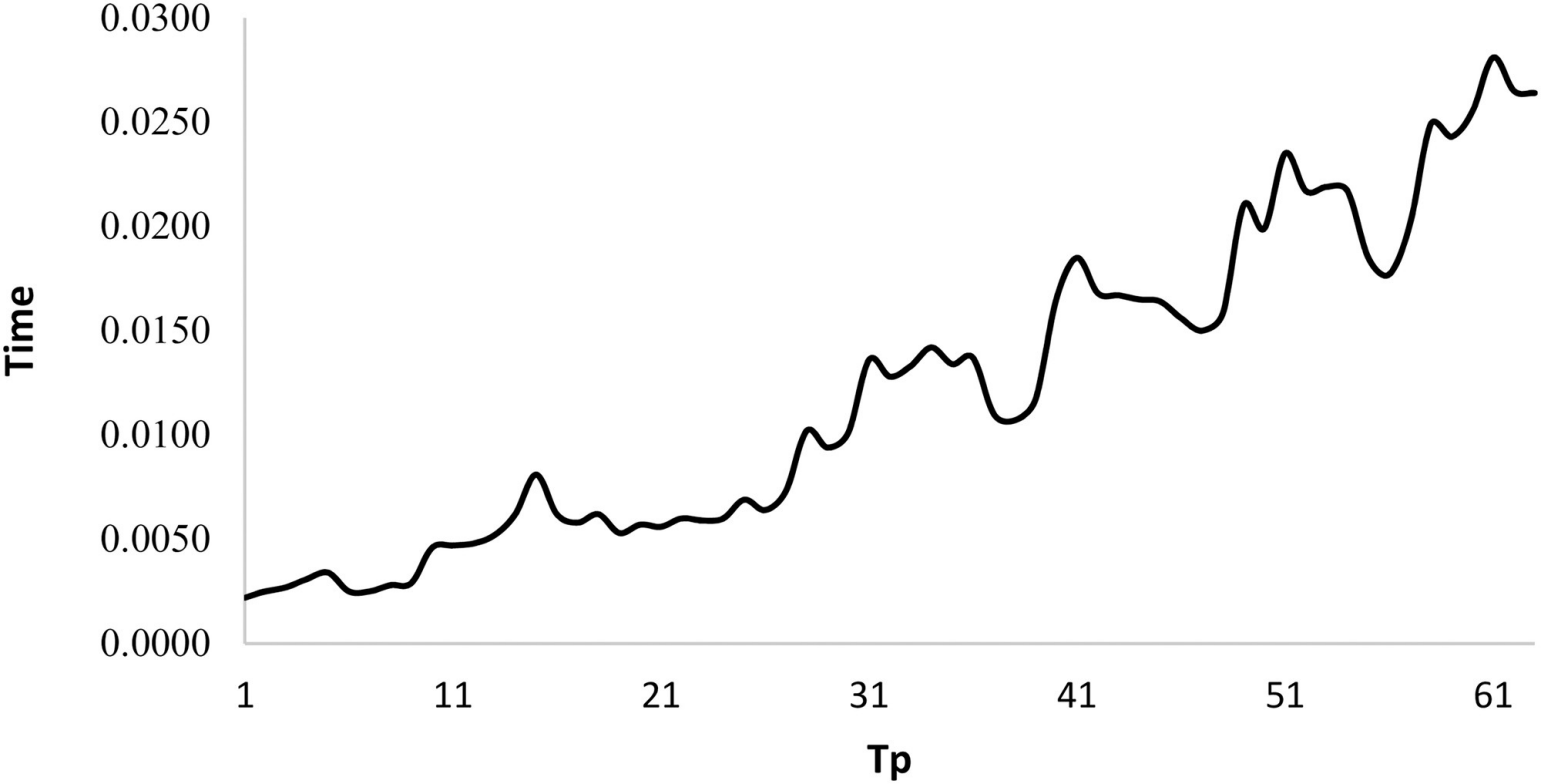
Time variation when *Tp* changes for algorithm *BPC*.

Fig 7 shows the *Time* behavior when *Tp* changes for *BPC*. This figure prove that the *Time* values are constantly increasing when *n*_*pr*_ increase. The maximum *Time* value of 0.0281 s is returned where (*n*_*pr*_, *n*_*su*_, *class*) = (100, 14, 1) and the minimum *Time* value of 0.0022 s is returned where (*n*_*pr*_, *n*_*su*_, *class*) = (10, 4, 1).

### 6.3 Discussions

Seven algorithms are proposed and tested in this paper. Table 3 shows the overview of the results given by all algorithms. this table shows that the best algorithm that reached the maximum percentage is *BPC* with a percentage of 72.86%. This percentage is not reaching the value of 100%, this is means that there is no dominance between the proposed algorithms. Indeed, for certain instances, the other algorithms excepting *BPC* give best results than *BPC* but in total *BPC* reaches 72.86%. The algorithms *PCP*, *NCP*, and *DCP* give closer results in the range of 25%. The maximum average time obtained by all algorithms is around 0.0121 s. This gives a remarkable runtime to reach an efficient solution. It is worth noting that, the *TVP* reached a 45.08% as performance compared with *BPC* that has 72.86% as a performance, in the same average runtime of 0.0121 s. Therefore, by consuming the same time a better solution can be reached by choosing the algorithm *BPC*. In this paper, the choice of three classes is based on several works in literature that use these classes and the generation of the instance to test the obtained results. Indeed, the choice is to give a variety of different ranges of the scale of the proposed problem. For Class 1, the range is for the small instances. For Class 2, the range is for the medium instances. Finally, Class 3 is for the big-scale instances reaching 500 programs. On other hand, a different range of scales is proposed for the number of programs and the number of supercomputers. The runtime of the algorithms is related to the structure of the utilization of the loop instructions in each algorithm. For *LTA* and *STA*, the average execution time is always less than 0.0001 s. This is obtained because these algorithms utilizing the dispatching-rules which are executed by calling the heap-sort algorithm which is *O*(*nlogn*). However, for algorithms *PCP*, *NCP* and *DCP* the complexity is *O*(*n*^2^). This explains the average execution time which is very close.

The algorithms proposed in this paper are based on several variants of the probabilistic method. In practice, the probabilistic method and the randomization approach provide a better solution for the scheduling problem. This is due to the multiple choice selection of the programs and the repetitive procedure. This observation is always applicable for the studied problem when the best-programs choosing algorithm is the best compared with other algorithms. Consider the two case studies for the presented problem: the small-scale instances and the big-scale instances. The small-scale instances are the instances such as *n*_*pr*_ ≤ 50. However, the big-scale instances are the instances such as *n*_*pr*_ > 50. The *BPC* algorithm gives remarkable results for the first case study which is the small-scale instances and for the second case study which is the big-scale instances in term of gap. Indeed, the average gap of all small-scale instances is 0.0616 and the average gap of all big-scale instances is 0.0451. In term of running time, the *BPC* algorithm gives remarkable results for the first case study and for the second case study. Indeed, the average running time of all small-scale instances is 0.0067 and the average running time of all big-scale instances is 0.0194. The proposed algorithms can be applied on several real applications. Indeed, the parallel computing which is a type of computation in which several calculations must be executed at the same time. Big problems can be classed into many small problems, which may then be solved simultaneously. Other real application of the proposed algorithms is the parallel programming which is the process of utilizing a group of resources to solve a problem in minimum time by partitioning the big work. It is worth noting that the studied problem is an NP-hard one. Consequently, a good approximate solution represents a great archive for the studied problem. Several meta-heuristics can be applied using the proposed algorithms to enhance the obtained results.

## 7 Conclusion

This paper discussed the program’s planning problem. These programs must be executed by a fixed number of available supercomputers. Each program requires a long period of time to be executed. the presented problem is strongly very hard. Especially, the hardness of the problem appears face on a big number of programs. In this paper, seven algorithms were developed to present solution of the studied problem. These algorithms utilize the dispatching rules and the randomization approach with several variants. The experimental results show that an efficient feasible solution can be offered in an acceptable running time. The best-developed algorithm is the best-programs choosing algorithm in 72.86% of instance cases. In addition, the experiments show that the developed algorithms do not impose any dominance on them.

From a perspective of the studied problem, the developed algorithms can be exploited to develop a new better solution based on metaheuristics like a genetic algorithm or swarm optimization. The optimal of the studied problem can be constructed by an exact solution method based on the tree searching. In this case, the developed algorithms will be used to test and calculate certain rules in the node of the research tree.
